# The Institutional Library

**Published:** 1921-09-24

**Authors:** 


### THE INSTITUTIONAL LIBRARY.

**Electro-Therapeutics for Practitioners.** Second edition. By F. Howard Humphriss. (Oxford Medical Publications. Price 21s. net.)

The second edition of this book has been considerably enlarged, and the material formerly included has been to a great extent re-written, so that the volume is now thoroughly up to date.

The addition of a chapter on radio-therapy does not add to the value of the book; it is impossible to deal in any way adequately with radio-therapy as a small section of a text-book of electro-therapy, and the subject is so fraught with difficulties and potential dangers that unless discussed very fully it is much better excluded. In the present instance the details given are quite insufficient; nor can the author's digression into the realms of radiographic diagnosis be recommended. Another chapter which is new to this edition—" Galvanism and Faradism and the Sinusoidal Currents "—also errs on the side of undue brevity. The matter is well arranged, and, with the exceptions noted above, adequate detail is given of the various modalities and their methods of application. The book will be particularly valuable to those who have already had some practical introduction to electro-therapy, as it incorporates the results of the author's wide experience in this speciality.

**Gynæcology.** By Brooke M. Anspach, M.D. (J. B. Lippincott. 1921. Pp. 752. Price 42s.)

This comprehensive textbook includes a good deal (rightly, as we think) which is usually omitted from British monographs of similar aim : such as sections upon diseases of the bladder and kidneys (forty-seven pages), of the abdominal viscera (twenty-two pages), of the anus and rectum, and upon backache, with a dissertation upon corsets. Speaking generally—though with certain minor exceptions in regard to urinary surgery—these sections fully reflect the best teaching of British and American specialists in the matters dealt with; and it is useful for gynæcologists to be reminded that disease respects no anatomical or conventional boundaries, and that they ought to have what some of them in this country have not, a working knowledge of up-to-date surgery of the urinary and alimentary systems, as well as of that of the reproductive organs pure and simple.

The arrangement of the book is good, the teaching is clear and explicit, the illustrations are excellent, and there is a commendable absence of padding. It is gratifying to see the Percy treatment of inoperable carcinoma of the uterus expounded at length, though the account does not read as if the author has tried it himself; this method, recently the subject of a paper at the Royal Society of Medicine, has been too much neglected in this country. The technique of operative procedures is most carefully described; the author has evidently taken the greatest care to embody in his practice every novelty in technique as to the soundness of which he is convinced. This is a most satisfactory textbook, and should become very popular.

**A Text-Book of Special Pathology.** By T. Martin Beattie, M.A., M.D., and W. E. Carnegie Dickson, M.D., F.R.C.P. (Ed.). Second Edition. (London: Wm. Heinemann. Price 31s. 6d. net.)

The second edition of this well-known work is deserving of all praise. The whole is largely re-written, and in every way brought up to date. There are 267 excellent illustrations in the text, and four coloured plates drawn from original preparations. Those who have in the past used this text-book will appreciate our meaning when we say that the old standard has been perfectly maintained.

**String Figures: A New Amusement for Everybody.** By W. W. Rouse Ball, Fellow of Trinity College, Cambridge. (W. Heffer & Sons, Ltd., Cambridge. Pp. 69. Price 2s. 6d. net. 2nd edition. 1921.)

This unique little book is not one to be taken up merely with the object of whiling away a few idle minutes; the interest of its contents is too absorbing for that. Already the first edition of 1920 is exhausted, and in this one some more figures have been inserted, the diagrams illustrating the figures, which are all extremely good, being drawn as seen by the operator himself, this making them easier to imitate. To convalescent and chronic patients, long-distance travellers, and those interested in folk lore, the study of these string figures cannot fail to be interesting. They are considered first as an amusement of primitive man, then in the light of their historical and religious associations, their classification and varieties. At the end of the book is a list of the sources from which the figures described have been taken. These will prove helpful to those who desire to pursue further the study of this fascinating hobby. Most people in their nursery days have played "cat's-cradle." They will find the simple devices of "cat's-cradle" enlarged and glorified in this book, some of the figures being anything but easy to make. Their study and construction is an interesting and most inexpensive hobby, all that is needed for it being found in this little volume and a long piece of string.

**Pope's Manual of Nursing Procedure.** A. E. Pope. (G. P. Putnams Sons. $2.00.)

This book is intended to facilitate the teaching of theoretical nursing by demonstrations: an excellent method, especially for junior pupils. Most girls find it very hard to understand the right way of lifting patients, making special beds, or giving treatment, by description only; but their interest is roused by demonstrations, and they remember readily what they are shown. The different methods of nursing technique are clearly and tersely described, though here and there the American wording will require explanation to English pupils. Hygiene, anatomy, and physiology are taught in connection with nursing points, and their bearing on a nurse's work explained. By this method the pupil sees at once the use of these studies; under the old system of teaching them separately the majority of probationers found them very wearisome. The early sections of the book are devoted to routine ward work. The author remarks that it should not be necessary to teach women to dust; but all ward sisters will endorse her conclusion that the necessity exists. Every procedure is explained, and the reason for it given. It is intended that the pupils should study each section first, and should then see the methods demonstrated, and take turns to practise them, in class. The teacher also questions the whole class on the different subjects. This is called, in American, "holding a quiz." It will probably often become a quiz in the English sense of the word as well. One good suggestion in the later part of the book is that, even if the class has a "hospital doll" to practise on, the pupils should take turns to act as patient, that they may work on the living subject. The rules given for the comfort and mental rest of the patients are excellent, and will be very helpful to young nurses.

